# EasyDAM_V2: Efficient Data Labeling Method for Multishape, Cross-Species Fruit Detection

**DOI:** 10.34133/2022/9761674

**Published:** 2022-09-10

**Authors:** Wenli Zhang, Kaizhen Chen, Chao Zheng, Yuxin Liu, Wei Guo

**Affiliations:** ^1^Information Department, Beijing University of Technology, Beijing 100022, China; ^2^Graduate School of Agricultural and Life Sciences, The University of Tokyo, Tokyo 188-0002, Japan

## Abstract

In modern smart orchards, fruit detection models based on deep learning require expensive dataset labeling work to support the construction of detection models, resulting in high model application costs. Our previous work combined generative adversarial networks (GANs) and pseudolabeling methods to transfer labels from one specie to another to save labeling costs. However, only the color and texture features of images can be migrated, which still needs improvement in the accuracy of the data labeling. Therefore, this study proposes an EasyDAM_V2 model as an improved data labeling method for multishape and cross-species fruit detection. First, an image translation network named the Across-CycleGAN is proposed to generate fruit images from the source domain (fruit image with labels) to the target domain (fruit image without labels) even with partial shape differences. Then, a pseudolabel adaptive threshold selection strategy was designed to adjust the confidence threshold of the fruit detection model adaptively and dynamically update the pseudolabel to generate labels for images from the unlabeled target domain. In this paper, we use a labeled orange dataset as the source domain, and a pitaya, a mango dataset as the target domain, to evaluate the performance of the proposed method. The results showed that the average labeling precision values of the pitaya and mango datasets were 82.1% and 85.0%, respectively. Therefore, the proposed EasyDAM_V2 model is proven to be used for label transfer of cross-species fruit even with partial shape differences to reduce the cost of data labeling.

## 1. Introduction

High-performance visual perception systems (as a key technology for automated fruit operation system in orchards) can be applied to smart orchard such as fruit positioning [1, 2], orchard yield statistics [3, 4], and automatic fruit picking [5, 6], by combining intelligent mechanical equipment. However, most current visual detection techniques are based on strongly supervised models of deep learning [7–11] that rely on labeled datasets to support the model training. As a result of the weak generalization capability of deep learning models, the application of fruit detection models in different scenarios (including same species in different origins, different species, and different weather and light condition) requires reconstructing new fruit datasets and training new detection models, which is time-consuming and labor-intensive. Meanwhile, the dense growth of fruit, the occlusion and shading between fruits and branches, and the small size of fruit in the actual orchard make the fruit labeling work more challenging. Therefore, there is an urgent need for a method that can effectively reduce the cost of fruit labeling.

In our previous research [12], a cross-species fruit dataset label conversion method (EasyDAM) was proposed by combining unsupervised image translation techniques and a pseudolabel method. This was applied to the label conversion between different fruit species but with similar shapes (e.g., orange to apple and orange to tomato), thus, saving fruit labeling cost. However, there were two main limitations of the EasyDAM: (1) the current unsupervised image translation model mainly realized the migration of color and texture features but with difficulties in shape features between source and target domain, which affects the quality of the obtained synthetic fruit images, and (2) pseudolabel method requires a large number of experiments to determine the confidence thresholds for detection models manually. Therefore, this paper is aimed at further investigating the label transfer methods to overcome these drawbacks of EasyDAM.

With the rapid development of GAN (generative adversarial network) [13] technology, its research techniques in image translation are often applied to solve the shortage of training image data in deep learning. Most current works [14–16] can achieve color and texture feature migration between different image domains. The shape feature, an important appearance feature of objects in images, can be used as a feature basis for detection in synthetic images generated by GAN. Some scholars have proposed introducing supervised signals of object shape feature information to supervise the training of GAN. Mo et al. [17] proposed Insta-GAN that jointly encodes image features and corresponding object instance mask attribute features to translate foreground object instances and retain original image background information. This could effectively solve the current difficulty in image translation with significant differences in object shapes. Chen et al. [18] adapted variational inference to disentangle the shape and appearance of the given images. This could generate person images with arbitrary shapes and allow the user to manipulate the degree of deformation of the rendered image explicitly. Liang et al. [19] proposed the Contrast-GAN by combining the information of instance masks that control the translation of features such as the shape and texture of object instances, by learning the semantic content information of image foregrounds (in different domains). Roy et al. [20] proposed the Segmantic-Aware-GAN that uses the object instance mask information as a supervised signal to learn foreground object features, and it further adopts the cross-domain semantic consistency loss function to retain the geometric structure semantic information. From the above research results, it can be seen that most of the supervised image translation models rely on the object instance mask to supervise the training of the models. However, the image object instance mask leads to a large and costly labeling workload, making them difficult to apply to practical work tasks.

Therefore, some scholars have also researched effectively translating the object shape feature under unsupervised GAN conditions. Wu et al. [21] proposed a disentangle-and-translate framework to handle the image translation task that encourages the network to learn independently but complements representation information by introducing the geometric loss and conditional variational autoencoder (VAE) loss. This is done while decomposing the image space into Cartesian products of the appearance and geometry latent spaces to learn the image mapping relationships between different domains. Zhao et al. [22] proposed an adversarial consistency loss and combined it with other loss functions to optimize the image translation model that could effectively preserve the original image semantic content information and apply it to object shape translation. Kim et al. [23] assisted the network in distinguishing image feature differences by introducing an attention module to control the feature variables of the shape and texture in the generated images and guide the network to translate the semantically important image regions. Nizan and Tal [24] used multiple sets of generators and discriminators trained in conjunction and output consistent features based on the multiple sets of generator networks. This avoided the constraints on shape changes during image translation by traditional cycle consistency loss methods. By analyzing the advantages and disadvantages of different existing image translation networks, Gokaslan et al. [25] proposed the GAN-imorph, which could be effectively applied to the image domain translation tasks with significant shape differences. Most of the above research works achieved translation with a single-object category and simple background (it is difficult to achieve image translation in complex scenes). However, implementing cross-species fruit image translation with shape differences under unsupervised conditions is also a difficult task that needs further addressed in this study.

In addition, during the use of the current pseudolabel method [26–29] for object detection, the accuracy of the generated label data depends not only on the detection performance of the model but also on the detection model confidence threshold setting. Wang et al. [30] proposed a cross-domain object detection model based on coteaching, where different confidence thresholds were manually set in different datasets to obtain a pseudolabel, which was applied to construct an object detection model in different domain street scenes. Wang et al. [31] used the confidence score of the detection boxes to measure the image global region uncertainty, a combination of the background region similarity and overlap degree, to measure the image local region uncertainty. This was done in order to solve the noisy pseudolabel overfitting problem by manually setting the confidence threshold to obtain a high-quality pseudolabel. Liu et al. [28] proposed a category-balanced loss function to optimize the detection model to alleviate the problem of pseudolabel bias caused by category imbalance; the model was then applied to traverse different values as confidence thresholds in the range of 0 to 0.9 with an interval of 0.1, and the experimental results under different confidence threshold conditions were compared. Ramamonjison et al. [32] proposed a new data augment method and teacher-guiled gradual adaptation method to reduce the impact of generating noisy label data during different domain adaptations. They did not take into account the impact of the confidence threshold on the quality of the final pseudolabel generation. Yang et al. [33] updated the generated pseudolabel by combining the historical predicted pseudolabel information and the nonmaximum suppression (NMS) method in order to eliminate the uncertainty of the generated pseudolabel during different iterations. The experimental results were compared under different confidence threshold conditions to select the best experimental results as the output. For the above research work and most other pseudolabeling works [26, 27, 34, 35], the confidence threshold is usually based on manual empirical values to obtain the final label data. This makes it difficult to objectively select the optimal confidence threshold values based on the model performance and the complexity of the dataset itself. Ignoring the impact of the model confidence threshold on the quality of the final generated label data and having a large number of different confidence threshold comparison tests lead to low efficiency in the method application.

Therefore, we propose EasyDAM_V2 to achieve efficient label conversion between different species of fruit datasets with partial shape differences. In this method, based on the original CycleGAN [14], we propose a multilayer feature fusion strategy in the generator network, a multidimensional feature loss function for fruit images, and a cross-cycle loss function comparison path to design a new unsupervised cross-species fruit image translation model (called the Across-Cycle Generative Adversarial Network (Across-CycleGAN)). The model can be applied to cross-species fruit image transformation tasks with partial shape differences and the construction of target domain pretrained fruit detection models. In addition, based on the constructed target domain pretrained fruit detection model, we further propose a pseudolabel adaptive threshold selection strategy. This can automatically calculate and adjust the confidence threshold values of the fruit detection model by measuring the pseudolabel quality characteristics (including quantity and score information) generated under different confidence threshold conditions. It can efficiently realize the conversion of label data between different species of fruit datasets and improve the application efficiency of the pseudolabeling method. The method proposed in this study leads to the following main contributions:

1. To address the translation problem for the different species of fruit images with partial shape differences, this paper proposes a new unsupervised fruit image translation network called Across-CycleGAN. The Across-CycleGAN does the following: it enhances the extraction ability of global shape features by introducing a multilayer feature fusion strategy in the generator network. Further, the paper proposes a multidimensional feature loss function that uses a cross-cycle loss function comparison path to learn the shape feature of fruit images interactively, effectively trains the network to learn shape feature differences between different species of fruit images, and generates synthetic fruit images suitable for deep learning detection model training
2. To address the problem of manually determining the confidence threshold in the pseudolabel process, this paper proposes a pseudolabel adaptive threshold selection strategy. This strategy measures the quality of the generated label data under different confidence thresholds and adaptively calculates the corresponding optimal confidence threshold for the pseudolabel

## 2. Materials and Method

**Figure 1:**
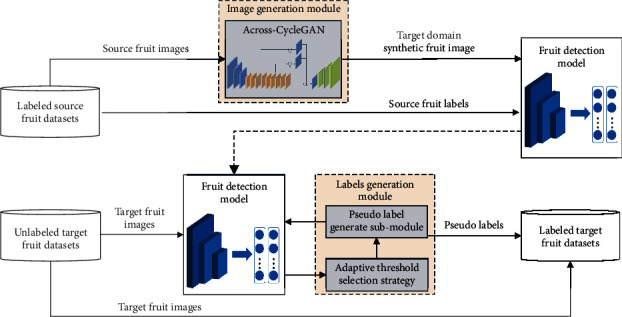
Flowchart of the cross-species fruit label conversion method EasyDAM_V2, including two main improvements (highlighted in the orange-colored boxes): (1) the use of the Across-CycleGAN in the image generation module, which can be applied to fruit image translation with partial shape differences, and (2) in the label generation module, an adaptive threshold selection strategy is used to calculate the optimal confidence threshold and obtain the pseudolabel automatically.

The flowchart of the proposed EasyDAM_V2 is shown in Figure 1, and each step is described as follows.

First, we input the labeled source domain fruit dataset, translate the source domain fruit image to the target domain synthetic fruit image by the fruit image translation model (in the image generation module), and construct the labeled target domain synthetic fruit dataset (by combining the label data of the source domain fruit dataset). The fruit detection model OrangeYolo [36] proposed in our previous research work is then applied to construct the target domain pretrained fruit detection model. In the image translation module, based on the original CycleGAN, this paper proposes the Across-CycleGAN (introduced in Section 2.2). The Across-CycleGAN is a fruit image translation network that can be applied to fruit images with partial shape differences (based on the improvement of both network structure and loss function) to generate the target domain synthetic fruit images suitable for deep learning detection model training.

Second, the unlabeled target domain actual fruit images are input into the constructed pretrained fruit detection model for the target domain. Then, the adaptive threshold selection strategy is used in the label generation module to select the optimal initial confidence threshold for the fruit detection model, and finally, it obtains the target domain actual fruit image pseudolabel (to construct the labeled target domain actual fruit dataset). In the label generation module, this paper proposes a pseudolabel adaptive threshold selection strategy (introduced in Section 2.3) that can automatically calculate the optimal confidence threshold of the fruit detection model and efficiently obtain the pseudolabel.

Finally, the labeled target domain actual fruit dataset constructed in the previous step is cycled into the pretrained fruit detection model of the target domain for fine-tuning. This is done while using the pseudolabel adaptive threshold selection strategy to adjust the confidence threshold dynamically and update the pseudolabel of the target domain actual fruit dataset. When the fruit detection model reaches a certain number of training epochs, it outputs the pseudolabel of the target domain actual fruit dataset. It then realizes the label conversion from the labeled source domain's fruit dataset to the unlabeled actual fruit dataset of the target domain.

This paper mainly introduces the fruit image translation network Across-CycleGAN in the image generation module and the pseudolabel adaptive threshold selection strategy in the label generation module.

### 2.1. Fruit Datasets

This study employed two datasets: the fruit image translation dataset and fruit detection dataset, which were applied for training the Across-CycleGAN and OrangeYolo models, respectively. The dataset mainly used orange fruit as the source domain dataset and pitaya and mango fruits as the target domain dataset. The fruit image translation dataset and the fruit detection dataset are described below.

#### 2.1.1. Fruit Image Translation Datasets

In the training process of the fruit image translation model Across-CycleGAN, two datasets, *orange&pitaya* and *orange&mango*, were mainly used to realize the image translation operation from orange to pitaya and from orange to mango, respectively. The fruit images in the dataset were searched from the Internet (without copyright restrictions), and the fruit image resolution was uniformly adjusted to a size of 256 × 256 to facilitate the input of the fruit image translation model for training.

1. *orange&pitaya* dataset: the dataset mainly contained fruit images of two species, orange and pitaya fruits. The training set contained 980 orange fruit images and 416 pitaya fruit images
2. *orange&mango* dataset: the dataset mainly contained fruit images of two species, orange and mango fruits. The training set contained 980 orange fruit images and 141 mango fruit images

#### 2.1.2. Fruit Detection Datasets

**Figure 2:**
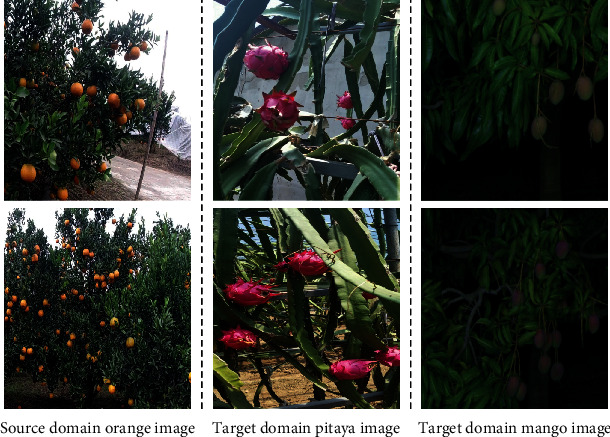
Different species of fruit images are shown in the actual orchard scene: the first column represents the source domain orange image; the second and third columns represent the target domain actual pitaya images and mango images, respectively.

In fruit detection, fruit images from actual orchard scenes were used to produce labeled fruit datasets for the subsequent method validation. The sample images of the fruit detection dataset are shown in Figure 2 and are described as follows:

1. Source domain orange dataset: the dataset follows the orange fruit dataset in the object detection dataset section of the EasyDAM [12] research. The orange images were mainly collected from an orange orchard in Sichuan (province), China. The fruit image acquisition equipment used a DJI Osmo action camera (Shenzhen DJI Technology Co., Ltd., China), and 664 orange images were collected, including images under various complex scenes, such as with occlusion and backlighting. These fruit images were manually labeled
2. Target domain fruit datasets: contained the pitaya and mango datasets: target domain pitaya datasets: the data was collected from an orchard in Beijing, China, using a Samsung Galaxy S8 phone (Samsung Electronics Technology Co., Ltd., South Korea) while the pitaya images in the orchard were additionally collected online and integrated. The dataset contained 377 pitaya images; the training set contained 265 unlabeled pitaya images, and the test set contained 112 labeled pitaya images

Target domain mango dataset: the dataset used the mango dataset published in research [37]. The fruit images were collected from a Queensland orchard in Australia under a dark scene using a Canon EOS 750 model camera (Canon Corporation, Japan). The dataset contained 620 mango images; the training set contained 516 unlabeled mango images, and the test set contained 104 labeled mango images.

### 2.2. Fruit Image Translation Network Across-CycleGAN

**Figure 3:**
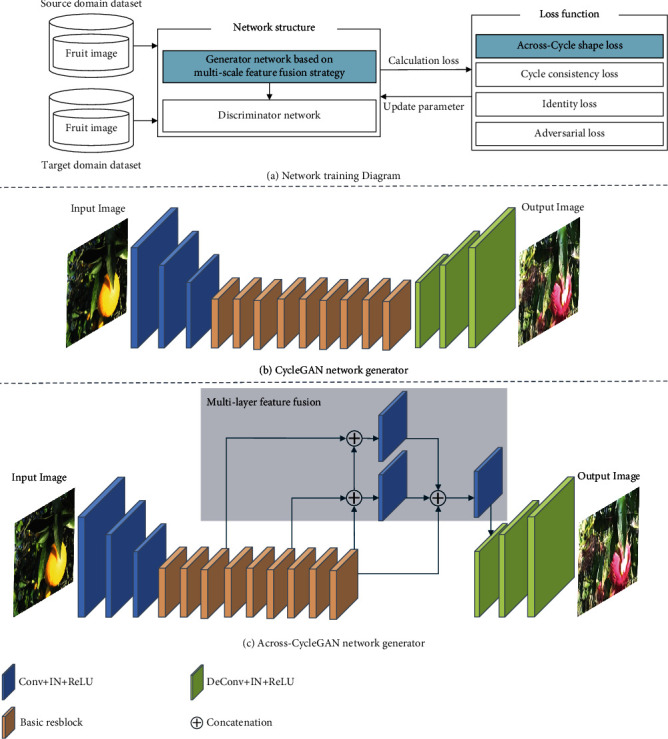
Training flowchart of fruit image translation network and comparison of the generator network structure: (a) the training flowchart of the fruit image translation network; (b) the generator network structure in the original CycleGAN; and (c) the generator network based on the multilayer feature fusion strategy.

The proposed Across-CycleGAN can be applied to fruit image translation with partial shape differences. The model training flow chart is shown in Figure 3(a). The improvement of the Across-CycleGAN mainly consists of the following: introducing a multilayer feature fusion strategy in the generator network (introduced in Section 2.2.1), constructing a multidimensional feature loss function (introduced in Section 2.2.2), and designing a cross-cycle loss function comparison path (introduced in Section 2.2.3). In this study, we predefined two image domains: the source domain *S* and the target domain *T*. The Across-CycleGAN mainly includes two generator networks: *G*_*S*−>*T*_ and *G*_*T*−>*S*_; and two discriminator networks: *D*_*S*_ and *D*_*T*_ (which aim to learn the image distribution mapping relationship between different image domains).

#### 2.2.1. Multilayer Feature Fusion Strategy

In this paper, we propose a multilayer feature fusion strategy in the generator network to fuse the image feature of the deep and shallow network layers to enhance the network to learn the shape feature of the fruit image.

We use the image translation process from the source domain to the target domain as an example. In the generator network of the original CycleGAN (shown in Figure 3(b)), the source domain image features are first extracted by the convolutional layer module. Then, the residual network module is used to learn the image distribution mapping relationship between different image domains, which can effectively preserve the original image feature information. This paper proposes a multilayer feature fusion strategy in the generator network to accommodate the translation of different species of fruit images with partial shape differences (as shown in Figure 3(c)). It extracts image features from different depth network layers and effectively enhances the fusion of deep and shallow image feature information to improve the quality of the features extracted by the network. The deep layer has a large receptive field that is beneficial for the network to learn the global shape feature information of the fruit. The shallow layer has a small receptive field that usually learns the local color and texture feature information of the fruit. Finally, the features extracted by the network are upsampled by the deconvolution layer module, and the target domain synthetic fruit image is output.

Meanwhile, the discriminator network follows the PatchGAN [38] in the original CycleGAN, which is a fully convolutional network consisting mainly of five convolutional layer modules. The PatchGAN network adopts a way of discriminating each image block region of the input image individually, effectively focusing on more local detail information in the image, and giving more accurate discriminative information.

#### 2.2.2. Multidimensional Feature Loss Function Design

**Figure 4:**
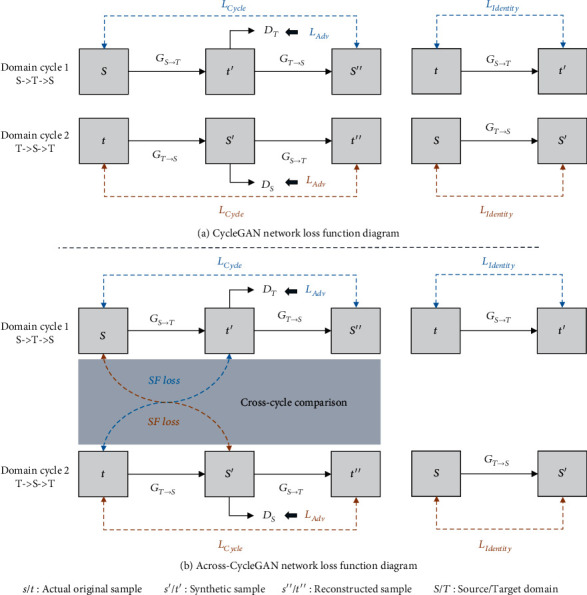
Schematic of the loss functions in different image translation networks: (a) the loss function of the original CycleGAN and (b) the loss function of the Across-CycleGAN proposed in this paper.

In this paper, we propose introducing shape feature loss functions based on the original CycleGAN and constructing multidimensional feature loss functions for color, texture, and shape features (to train the fruit image translation network). In the original CycleGAN (shown in Figure 4(a)), a combination of the cycle consistency loss function (*L*_Cycle_) and identity loss function (*L*_Identity_) are used to ensure that the network can migrate the color and texture features between different species of fruit images under unsupervised and unpaired datasets conditions. At the same time, the adversarial loss (*L*_Adv_) is used to stabilize the network training effect and generate higher quality synthetic fruit images.

For the translation of fruit images with partial shape differences, due to the lack of fitting learning of fruit shape features during the training of the original CycleGAN, it will be difficult for the network to translate the shape features between different species of fruit images. Therefore, we propose to introduce a shape feature loss function (*L*_SF_) and then a multidimensional feature loss function constructed to optimize the network (as shown in Figure 4(b)). The overall loss function of the Across-CycleGAN is shown in Equation (1) as follows:

(1) $$\begin{array}{c} L_{\text{total}}=w_{\text{SF}}\times L_{\text{SF}}\left(G_{\text{ST}},G_{\text{TS}}\right)+w_{\text{Cycle}}\times L_{\text{Cycle}}\left(G_{\text{ST}},G_{\text{TS}}\right)+w_{\text{Identity}}\times L_{\text{Identity}}\left(G_{\text{ST}},G_{\text{TS}}\right)+w_{\text{Adv}}\times L_{\text{Adv}}\left(G,D,S,T\right), \end{array}$$

where *L*_SF_ is the shape feature loss function, *L*_Cycle_ is the cycle consistent loss function, *L*_Identity_ is the identity loss function, and *L*_Adv_ is the adversarial loss function, which together form the multidimensional feature loss function *L*_total_. The corresponding weight coefficients of each loss function are *w*_SF_ = 0.2, *w*_Cycle_ = 0.8, *w*_Identity_ = 1.0, and *w*_Adv_ = 1.0, which are applied to the network to balance the learning of different features. Each loss function is described as follows:

1. Shape feature loss

For the fruit shape feature loss function design, this paper proposes to introduce the shape feature loss function *L*_SF_, as described by Equation (2). The loss function is based on the multiscale structural similarity index method (MS-SSIM) [39], using different sized convolution kernels to adjust the image receptive field and statistical information on the structure features of the corresponding image regions (under different scale conditions). This can effectively distinguish the geometric differences between different species of fruit images and train the model to better adapt to the variation of fruit shape features. The shape loss function is calculated as follows:

(2) $$\begin{array}{c} L_{\text{SF}}\left(G_{\text{ST}},G_{\text{TS}}\right)=\left(1-\text{MS}-\text{SSIM}\left(G_{\text{ST}}\left(s\right),t\right)\right)+\left(1-\text{MS}-\text{SSIM}\left(G_{\text{TS}}\left(t\right),s\right)\right). \end{array}$$

(2) Cycle consistency loss

The cycle consistency loss function *L*_Cycle_(*G*_ST_, *G*_TS_) in the original CycleGAN is followed, which can effectively preserve the original image content information by limiting the size of the image distribution mapping space in different image domain translation directions. The correlation loss function is described by Equation (3) as follows:

(3) $$\begin{array}{c} L_{\text{Cycle}}\left(G_{\text{ST}},G_{\text{TS}}\right)=E_{s\sim \text{pdata}\left(s\right)}{\left\|G_{\text{TS}}\left(G_{\text{ST}}\left(s\right)\right)-s\right\|}_{1}+E_{t\sim \text{pdata}\left(t\right)}{\left\|G_{\text{ST}}\left(G_{\text{TS}}\left(t\right)\right)-t\right\|}_{1}. \end{array}$$

(3) Identity loss

The identity loss function *L*_Identity_(*G*_ST_, *G*_TS_) is used to train the network to learn a single mapping relationship of the image distribution in different image domain translation directions. This is done in order to enhance the ability of the network to preserve image color and texture features. The correlation loss function is described in Equation (4) as follows:

(4) $$\begin{array}{c} L_{\text{Identity}}\left(G_{\text{ST}},G_{\text{TS}}\right)=E_{s\sim \text{pdata}\left(t\right)}{\left\|s-G_{\text{ST}}\left(s\right)\right\|}_{1}+E_{s\sim \text{pdata}\left(t\right)}{\left\|t-G_{TS}\left(t\right)\right\|}_{1}. \end{array}$$

(4) Adversarial loss

The adversarial loss function *L*_Adv_(*G*, *D*, *S*, *T*) in the original CycleGAN is followed, including two parts of loss functions *L*_Adv_(*G*_ST_, *D*_*T*_, *S*, *T*) (as described in Equation (5)) and *L*_Adv_(*G*_TS_, *D*_*S*_, *S*, *T*), with the aim of stabilizing the network training effect and obtaining higher quality generated image results. The adversarial loss function *L*_Adv_(*G*_ST_, *D*_*T*_, *S*, *T*) is calculated as follows, as well: i.e., *L*_Adv_(*G*_TS_, *D*_*S*_, *S*, *T*):

(5) $$\begin{array}{c} L_{\text{Adv}}\left(G_{\text{ST}},D_{T},S,T\right)=E_{t\sim \text{pdata}\left(t\right)}\left[{\left(D_{T}\left(t\right)-1\right)}^{2}\right]+E_{s\sim \text{pdata}\left(s\right)}\left[D_{T}{\left(G_{\text{ST}}\left(s\right)\right)}^{2}\right]. \end{array}$$

#### 2.2.3. Cross-Cycle Loss Function Comparison Path Design

In the original CycleGAN, based on two generator networks (*G*_*S*−>*T*_ and *G*_*T*−>*S*_) and discriminator networks (*D*_*S*_ and *D*_*T*_), the different feature loss errors in two independent and different domain cycle directions (i.e., domain cycle 1: *S* > *T* > *S* and domain cycle 2: *T* > *S* > *T*) are calculated. However, during each domain cycle, for example, domain cycle 1: *S* > *T* > *S*, the domain cycle (shown in Figure 4(a)) translates the input source of the domain actual fruit image into the synthetic fruit image of the target domain through the generator network and reconstructs it back into the source domain fruit image. The loss errors in shape features of synthetic fruit images in the target domain and the actual fruit images in the target domain are not compared, which makes it difficult for the network to fit the actual fruit image shape features effectively (domain cycle 2: *T* > *S* > *T*).

Therefore, this paper proposes a hypothesis that the actual fruit shape features are used to train the network to fit the generated synthetic fruit image during the training process of different domain cycles. This helps the network to learn information about the difference in fruit shape features between different domains. Based on the assumption above, this paper proposes to design a cross-cycle feature loss comparison path in two independent and different domain cycle processes, which is applied to the calculations of the shape feature loss function errors (of different species of fruit images), as shown in Figure 4(b). During two different domain cycles, this method interactively learns the shape feature information of fruit images by calculating the shape feature loss error between the synthetic fruit in the current domain cycle and the actual fruit in the other domain cycle. It can effectively train the network to fit actual fruit shape features and generate synthetic fruit images suitable for deep learning detection model training.

### 2.3. Pseudolabel Adaptive Threshold Selection Strategy

In deep learning object detection tasks, the pseudolabel (similar to human-like data labeling) is automatically generated by the detection model, replacing the manual labor of labeling and effectively reducing the dataset labeling effort. The accuracy of the generated pseudolabel depends not only on the detection performance of the model but also on the size of the confidence threshold set by the detection model. When the confidence threshold of the model is set to a low threshold, the detection box retention condition is loose, and the number of acquired pseudolabels is high. This is to comprehensively cover the foreground object area in the image and provide more correctly labeled boxes for the detection model to train and retain more noisy label data (false detection boxes), i.e., in the case of pseudolabel generated under high confidence threshold conditions. The confidence threshold of the model is then analyzed in the process of pseudolabel generation. It is often necessary to find a balance point threshold in the process of setting the confidence threshold, which can make the generated pseudolabel balanced in terms of the quantity and confidence score (reduces the influence of noisy labels). However, in pseudolabeling methods proposed in previous research (EasyDAM [12]), a manual empirical value approach is usually used to set the confidence threshold and obtain the image pseudolabel, and finally, the best quality pseudolabel is filtered by comparing the experimental results under different confidence threshold conditions. However, in this method, it is difficult to select the best confidence threshold for a pseudolabel effectively, and the workload of the compared experiments under different confidence threshold conditions is large, resulting in low efficiency of the overall method application.

Therefore, this paper proposes an adaptive threshold selection strategy for pseudolabel generation methods by combining the fruit detection model OrangeYolo [36] proposed in our previous research work. The strategy, based on the target domain pretrained fruit detection model constructed by Across-CycleGAN, calculates the quantity and score information characteristics of the pseudolabel generated under the corresponding confidence threshold conditions to obtain the quality variance values of pseudolabel. Then, the confidence threshold corresponding to the maximum variance value is used as the confidence threshold balance point (i.e., the optimal confidence threshold) for the pseudolabel, which is able to dichotomize the pseudolabel into high- and low-quality categories of labels and maximize their differentiation. In the next step, high-quality category pseudolabels are selected to train the subsequent fruit detection model that adjusts the confidence threshold dynamically and updates the generated pseudolabel (by using the strategy method). This is done to gradually improve the performance of the fruit detection model and the accuracy of the generated pseudolabel. The strategy method is shown in Algorithm 1, and the implementation steps are described below:

1. Set the confidence threshold range *R* ∈ [0.10, 1.00], and obtain the total number *N* and the average confidence score *μ* of the pseudolabel within the confidence threshold range *R*
2. In the confidence threshold range *R*, iterate the different confidence threshold values at intervals of 0.01 and note the current confidence threshold value as Conf.
3. Count the confidence scores of the pseudolabels less than Conf. The number of pseudolabels and average confidence scores are denoted as *N*0 and *μ*0, respectively. Calculate the corresponding percentage of the number of pseudolabels *w*0 = *N*0/*N*.
4. Count the confidence scores of the pseudolabels greater than Conf. The number of pseudolabels and the average confidence scores are denoted *N*1 and *μ*1, respectively. Calculate the corresponding percentage of the number of pseudolabels *w*1 = *N*1/*N*.
5. Calculate the quality variance value *g*_Conf_ of the pseudolabel under the corresponding confidence threshold condition according to the following equation: *g*_Conf_ = *w*0(*μ*0 − *μ*)^2^ + *w*1(*μ*1 − *μ*)^2^.
6. Repeat steps (2)~(5) to count the variance values of the pseudolabel quality under each confidence threshold, and finally, select the confidence threshold corresponding to the maximum variance value as the best confidence threshold of the pseudolabel method

In the above method, the impact of differences in the quantity and score information of the generated pseudolabel under different confidence thresholds is measured, and the confidence threshold of the fruit detection model is adjusted dynamically. This enables to effectively reduce the negative impact of noisy labels in the subsequent fine-tuning of the fruit detection model. Meanwhile, this pseudolabel method can reduce a large number of comparison test work under different confidence thresholds, which improves the efficiency of the pseudolabel method application and obtains a high-precision pseudolabel.

### 2.4. Experimental Evaluation Metrics

To verify the accuracy of the labels generated from the target domain actual fruit dataset, the effectiveness of the proposed method was indirectly verified by the performance of the target domain fruit detection model obtained from the final training. The evaluation methods of target domain fruit detection models mainly use the precision, recall, *F*1 score, and average precision (AP) metrics. Higher values of the corresponding indexes indicate better detection model performance. Among them, the precision, recall, and *F*1 score are taken from the model performance balance point (i.e., where the precision value is approximately equal to the recall value) and the details of the evaluation methods can be found in the previous work [12].

## 3. Result

In the experiments of this study, the label conversion functions for the unlabeled target domain actual pitaya and mango datasets were implemented on the basis of the labeled source domain orange dataset. It was divided into the following two parts of experiments:

1. Based on the fruit image translation network (Across-CycleGAN), the target domain pretrained pitaya and mango fruit detection models were constructed, denoted as *M*_pitaya_ and *M*_mango_, respectively (introduced in Section 3.1)
2. Based on the constructed target domain pretrained fruit detection model, the pseudolabel of the unlabeled target domain actual pitaya and mango datasets were obtained by combining the pseudolabeling strategy method proposed in this study, denoted as the *orange2pitaya* experiment and *orange2mango* experiment, respectively (introduced in Section 3.2)

### 3.1. Verifying the Validity of the Generated Synthetic Fruit Images

In this section of the experimental work, on the basis of the labeled source domain orange dataset, the Across-CycleGAN was used to produce the labeled target domain synthetic pitaya and mango datasets, which were applied to construct the target domain pretrained pitaya and mango fruit detection models (respectively denoted as *M*_pitaya_ and *M*_mango_). We indirectly verified the effectiveness of the target domain synthetic fruit images generated by the Across-CycleGAN through the performance of the constructed target domain pretrained fruit detection model.

Meanwhile, in order to verify the effectiveness of the Across-CycleGAN, the current image translation algorithms that could effectively handle the object shape translation problem were selected for comparison in this portion of the experiment (the unsupervised generative attentional networks with adaptive layer-instance (U-GAT-IT) [23] and council generative adversarial network (Council-GAN) [24] were selected, respectively). The U-GAT-IT network [23] uses an attention mechanism to assist the network in focusing on the foreground region of the image to learn the foreground object shape feature information. The Council-GAN [24] uses the consistency of image feature outputs by multiple generators to avoid the difficult translation of object shapes caused by cycle consistency loss methods.

Table 1 shows the test comparison results of the Across-CycleGAN algorithm with other image translation algorithms, and the experimental results are analyzed and discussed. In the experiments of the target domain pretrained pitaya detection model *M*_pitaya_ (constructed based on the Across-CycleGAN), an AP value of 72.8% and an *F*1 score value of 71.0% were obtained from the test in the target domain of actual pitaya images. In the two-test metrics: the AP and *F*1 scores of the Across-CycleGAN test results improved by 5.3% and 3.7% as compared to that of the U-GAT-IT network [23]. It also improved by 61.8% and 50.5% compared to the Council-GAN [24]. Meanwhile, the target domain pretrained mango detection model *M*_mango_ (constructed by the Across-CycleGAN) was tested on the target domain of actual mango images in real scenes to obtain an AP value of 68.7% and an *F*1 score value of 67.8%, which were better than the experimental results of other images translation algorithms.

**Figure 5:**
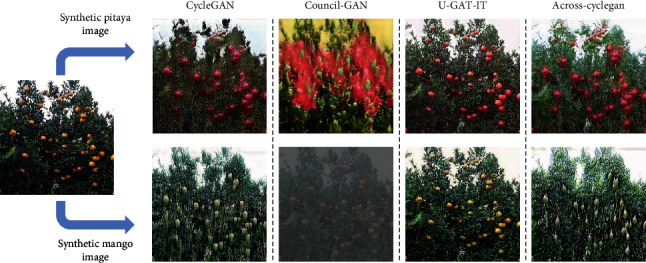
Target domain synthetic pitaya and mango fruit images generated by different image translation networks.

Figure 5 shows the target domain synthetic pitaya and mango images generated by different image translation networks. In the Council-GAN experiment [24], the generated target domain synthetic pitaya image was more seriously distorted, resulting in the background color features in the synthetic pitaya image being more similar to the foreground color features of the actual pitaya image (both presenting a red color). Most of the foreground color and texture features of pitaya image were not well translated. The fruit detection model was easy to learn the red color features of the background in the synthetic pitaya image as negative samples in the training process, resulting in poor performance of the final trained target domain pretrained pitaya detection model *M*_pitaya_. The AP value only reached 11.0%. Meanwhile, it was observed in the target domain synthetic mango images acquired by the Council-GAN [24] that their global image styles were translated to approximate the foreground feature styles of target domain actual mango image, which could ensure that the fruit detection model learned similar color and texture features of actual mangos. In addition, in the target domain synthetic pitaya image generated by the U-GAT-IT network [23], although there was no major change in the fruit shape, there was a correct translation of the color and texture features in the foreground of the fruit, so that the target domain pretrained pitaya detection model *M*_pitaya_ could be guaranteed to detect the actual pitaya images (based on the color and texture features). Meanwhile, in the target domain synthetic mango images obtained by the U-GAT-IT network [23], the color and texture features were translated to a lesser extent, while the local edge features were similar between different species of fruits, which caused the target domain pretrained mango detection model *M*_mango_ to still have preliminary mango fruit detection performance.

Finally, the results of different species of synthetic fruit images generated by the proposed Across-CycleGAN showed that the network could achieve the translation of foreground fruit color and texture features (while minimizing the distortion in the background region of the image). It also got a certain degree of translation in the shape features, bringing accuracy improvements with the best performance.

To further validate the effectiveness of each improved strategy in the proposed Across-CycleGAN, including the multilayer feature fusion strategy (MFFS) and the multidimensional feature loss function (MFL), relevant ablation experiments were conducted in this study. As shown in the experimental results in Table 2, based on the original CycleGAN, with the introduction of the MFFS and MFL, respectively, the network achieved a certain improvement in AP values compared to that of the original CycleGAN. It achieved the best AP values in the CycleGAN+MFFS+MFL (i.e., the Across-CycleGAN) experiment, which verified the effectiveness of each improved strategy in the Across-CycleGAN proposed in this study.

**Figure 6:**
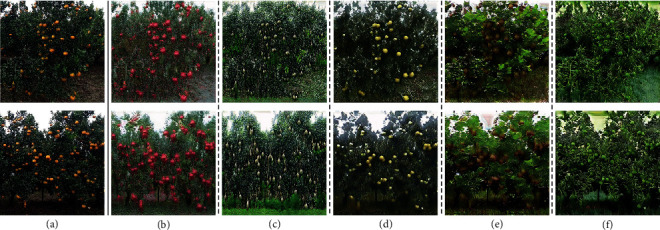
Real fruit images and the several synthetic fruit images generated by the Across-CycleGAN: (a) real orange images; (b–f) synthetic fruit images: pitaya, mango, pear, kiwi, and green pepper, respectively.

Meanwhile, we generated several other species of target domain fruits to validate the Across-CycleGAN effectiveness, with orange as the source domain fruit (containing 980 images) and pear, kiwi, and green pepper as the target domain fruits (containing 358, 597, and 391 images, respectively). The fruit images in the dataset were searched from the Internet (without copyright restrictions). As shown in Figure 6, the Across-CycleGAN can achieve better generation results when there are partial shape differences between the target domain fruit and the source domain fruit, even there are large surface texture differences between them.

### 3.2. Verifying the Validity of the Adaptive Threshold Selection Strategy

In the target domain pretrained fruit detection models (*M*_pitaya_ and *M*_mango_) obtained based on the Across-CycleGAN construction, this study proposed a pseudolabel adaptive threshold selection strategy to further obtain the pseudolabel of the target domain actual pitaya and mango datasets, respectively (denoted as the *orange2pitaya* and *orange2mango* experiments).

**Figure 7:**
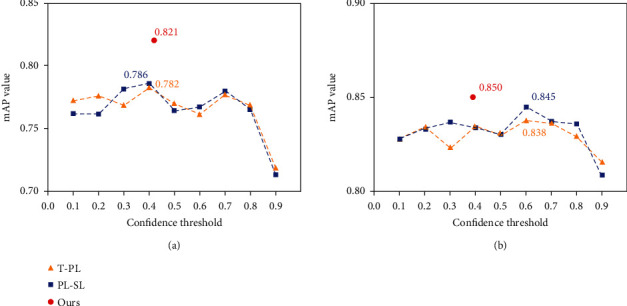
AP results for different pseudolabeling methods: (a) the results of the *orange2pitaya* experiment and (b) the results of the *orange2mango* experiment. T-PL denotes the traditional pseudolabeling method, PL-SL denotes the pseudolabel self-learning method, and ours denotes the pseudolabeling method proposed in this study.

In this section, the experimental results of the pseudolabel adaptive threshold selection strategy are mainly compared with those of the traditional pseudolabel method (denoted as T-PL) and the pseudolabel self-learning method (denoted as PL-SL) under different confidence threshold conditions, respectively (as shown in Figure 7). The traditional pseudolabeling method (denoted as T-PL) represents only a single acquisition of a pseudolabel to construct the labeled target domain actual fruit dataset and directly inputs to the fruit detection model for fine-tuning. The pseudolabel self-learning method (denoted as PL-SL) is the improved pseudolabeling method in our previous research work [12].

Table 3 shows the results comparing the proposed pseudolabel adaptive threshold selection strategy with other pseudolabel generation methods. The following experimental results are analyzed and discussed: in the *orange2pitaya* experiment, the initial optimal confidence threshold of the target domain pretrained pitaya detection model is 0.42, calculated using the pseudolabel adaptive threshold selection strategy. Meanwhile, during the fine-tuning process of the model, it adjusts the optimal confidence threshold adaptively and updates the pseudolabel dynamically. Finally, the AP value of the label data of the target domain actual pitaya dataset reaches 82.1% and the *F*1 score value reaches 78.0% in the experimental test, which is 3.9% higher than the best experimental results of the traditional pseudolabeling method T-PL (with a confidence value of 0.40). It is also 3.5% higher than that of the best experimental results of the pseudolabeling self-learning method PL-SL (with a confidence value of 0.40). In the *orange2mango* experiment, the initial optimal confidence threshold of the target domain pretrained mango detection model was 0.48 and the generated label data of the target domain actual mango dataset achieves AP and *F*1 score values of 85.0% and 81.7%, respectively. Compared with the traditional pseudolabeling method T-PL (with a confidence value of 0.60) and the pseudolabel self-learning method PL-SL (with a confidence value of 0.40), the AP values of our model improved by 1.2% and 0.5%, respectively, which are both better than the comparison algorithm experiments.

**Figure 8:**
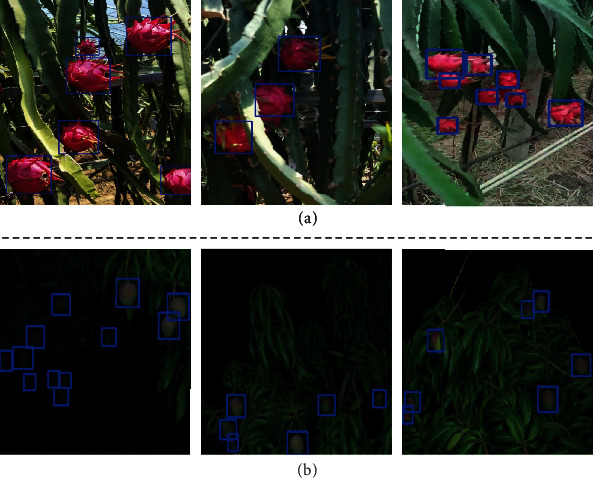
Visualization of the detection boxes for different species of fruit images in the actual orchard scene: (a) and (b) represent the detection results of actual pitaya image and actual mango image, respectively, where the blue rectangular box of the image represents the detection box. The detection box can be converted into image label data to realize the automatic labeling function of the fruit dataset.

Finally, the label data of the target domain actual pitaya and mango images generated by the proposed method in this study are visualized (as shown in Figure 8). In the *orange2pitaya* experiment, the target domain pitaya fruit showed irregular shape, which was different from the source domain orange fruit with a nearly round shape. The fruit images in the source and target domains are collected from two scenes: an outdoor orchard and an indoor greenhouse, respectively. From the experimental results, it is clear that for the task of the label conversion of fruit datasets with partial shape differences and large differences in scenes (indoor and outdoor), the label data generated by the proposed method in this study could correctly label the foreground pitaya fruit region of the image on a larger area. Meanwhile, in the *orange2mango* experiment, the target domain actual mango images were collected from the dark night environment when compared with the source domain orange images collected from a daytime environment. There were great differences in image scenes and illumination brightness. The method proposed in this study could achieve higher accuracy in the label conversion of the fruit dataset, which verifies the effectiveness of the proposed method.

## 4. Discussion and Conclusion

This study proposed a new cross-species fruit dataset label conversion method the EasyDAM_V2. The model can effectively apply cross-species fruit dataset label conversions with partial shape differences and improve accuracy and efficiency. It was applied to label conversions from the source domain orange dataset to the target domain of the pitaya fruit and mango dataset (to save the labeling work of the target domain fruit dataset).

**Figure 9:**
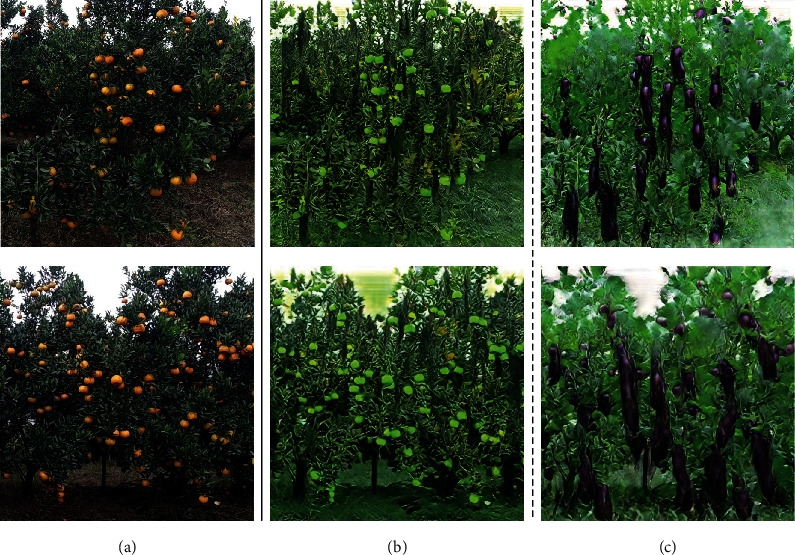
Fruit image translation with large differences in shape features by the Across-CycleGAN: (a) real orange images; (b) synthetic cucumber images, cannot present in the same position as the corresponding oranges; (c) synthetic eggplant images are not generated one-to-one with the oranges.

In the research of the Across-CycleGAN (the fruit image translation network), improvements were mainly made in both the network structure and loss function, which can be applied for translating different species of fruit images with partial shape differences. It was also compared with the current advanced image translation algorithms: the U-GAT-IT network [23] and Council-GAN [24], which can handle differences in shape between different domains. From the comparison of results with the two models, the effectiveness of the Across-CycleGAN proposed in this study was verified. At the same time, Across-CycleGAN is mainly used for different species of fruits with partial shape differences, while for fruit image translation with large differences in shape features (e.g., orange and cucumber and orange and long striped eggplant), the network needs to be further improved (to enhance the generalizability of the method). As shown in Figure 9, we can note that the synthetic cucumbers cannot present in the same position as the corresponding oranges, and the synthetic eggplants are not generated one-to-one with the oranges. In addition, the quality of the target domain synthetic fruit images affects the labeling accuracy of the final fruit dataset generation. The values of the weight coefficients assigned to different feature loss functions in the fruit image translation network affect the strength of the ability of the network to learn different image features and the quality of the generated target domain synthetic fruit images. Therefore, automatically assigning different loss function weight coefficients according to the feature differences among fruit images is also an improvement for future research on the fruit image translation network.

In the pseudolabel adaptive threshold selection strategy proposed in this study, the strategy combines the performance of the target domain pretrained fruit detection model and the image complexity of the unlabeled target domain fruit images (to automatically adjust the confidence threshold and obtain fruit image pseudolabel). The proposed pseudolabeling method was compared with the traditional pseudolabel and pseudolabel self-learning methods under different confidence threshold conditions. Meanwhile, the pseudolabel method proposed in this study mainly searches for the optimal confidence threshold balance points by measuring the quantity and score information characteristics of the pseudolabel. In order to reduce the negative impact of noisy pseudolabeling during the fine-tuning of the target domain fruit detection model, further filtering operations are required for the actual noisy label data in the generated pseudolabel. In addition, the target domain pretrained fruit detection model is mainly trained by the target domain synthetic fruit dataset. Both the synthetic fruit images and actual fruit images in the target domain have certain differences in the scale of the fruit, which may lead to the target domain pretrained fruit detection model in the pseudolabel generation process. Since the pseudolabel is more difficult to label the complete area of the foreground fruit accurately, this will result in the mislabeling phenomenon. Another future improvement in this study is to further solve the mislabeling phenomenon caused by the difference in fruit image scales between different domains.

In summary, the cross-species fruit dataset label conversion method proposed in this study for converting labels of different species of fruit datasets with partial shape differences can effectively solve the high cost of labeling fruit datasets problem. Meanwhile, in the application of modern intelligent orchards, according to the actual fruit detection task requirements, the method of this study can efficiently generate fruit image labels of the required species in the target task and quickly build a high-precision fruit detection model (solving the problem of complicated dataset labeling in the current deep learning fruit detection technology). It can be further equipped with relevant agricultural machinery and equipment applied to other intelligent orchard work to improve the intelligent efficiency of the orchard.

## Figures and Tables

**Algorithm 1:**
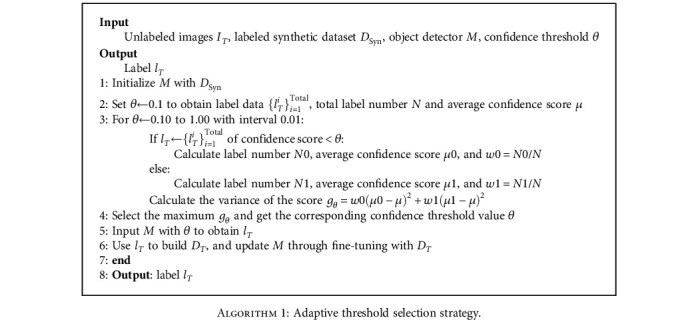
Adaptive threshold selection strategy.

**Table 1:** Test results of the target domain pretrained pitaya and mango fruit detection models constructed based on different image transformation networks.

Experiment	Model	Precision	Recall	*F*1 score	AP
*M*_pitaya_	U-GAT-IT	0.674	0.673	0.673	0.675
	Council-GAN	0.208	0.205	0.206	0.110
	CycleGAN	0.641	0.649	0.645	0.658
	**Across-CycleGAN**	**0.710↑**	**0.710↑**	**0.710↑**	**0.728↑**

*M*_mango_	U-GAT-IT	0.500	0.492	0.496	0.444
	Council-GAN	0.644	0.656	0.650	0.653
	CycleGAN	0.601	0.600	0.600	0.581
	**Across-CycleGAN**	**0.678↑**	**0.678↑**	**0.678↑**	**0.687↑**

**Table 2:** Across-CycleGAN ablation experiments.

Experiment	Model	Precision	Recall	*F*1 score	AP
*M*_pitaya_	CycleGAN	0.641	0.649	0.645	0.658
	CycleGAN+MFFS	0.679	0.680	0.679	0.664
	CycleGAN+MFL	0.691	0.693	0.692	0.702
	**Across-CycleGAN (CycleGAN + MFFS + MFL)**	**0.710↑**	**0.710↑**	**0.710↑**	**0.728↑**

*M*_mango_	CycleGAN	0.601	0.600	0.600	0.581
	CycleGAN+MFFS	0.597	0.601	0.599	0.651
	CycleGAN+MFL	0.644	0.639	0.642	0.660
	**Across-CycleGAN (CycleGAN + MFFSL + MFL)**	**0.678↑**	**0.678↑**	**0.678↑**	**0.687↑**

**Table 3:** On the basis of the target domain pretrained fruit detection model obtained from the Across-CycleGAN construction, the experimental test results of different pseudolabeling methods, namely, the T-PL and PL-SL, showed the best AP results amongst the corresponding pseudolabeling comparison methods.

Experiment	Pseudolabel method	Precision	Recall	*F*1 score	AP
*orange*2*pitaya*	T-PL	0.761	0.762	0.762	0.782
	PLSL	0.772	0.773	0.773	0.786
	**Proposed**	**0.782↑**	**0.778↑**	**0.780↑**	**0.821↑**

*orange*2*mango*	T-PL	0.799	0.795	0.797	0.838
	PLSL	0.812	0.813	0.813	0.845
	**Proposed**	**0.818↑**	**0.816↑**	**0.817↑**	**0.850↑**

## Data Availability

The data used in this paper will be available upon request here: https://github.com/I3-Laboratory/EasyDAM2.

## References

[1] Jian L., Mingrui Z., Xifeng G. A fruit detection algorithm based on r-fcn in natural scene.

[2] Ge Y., Xiong Y., From P. J. (2020). Symmetry-based 3d shape completion for fruit localisation for harvesting robots. *Biosystems Engineering*.

[3] Anderson N. T., Walsh K. B., Wulfsohn D. (2021). Technologies for forecasting tree fruit load and harvest timing—from ground, sky and time. *Agronomy*.

[4] Koirala A., Walsh K. B., Wang Z. (2021). Attempting to estimate the unseen—correction for occluded fruit in tree fruit load estimation by machine vision with deep learning. *Agronomy*.

[5] Yang Z. (2021). Research on the application of rigid-flexible compound driven fruit picking robot design in realizing fruit picking. *Journal of Physics: Conference Series. IOP Publishing*.

[6] Wang H., Zhao Q., Li H., Zhao R. (2022). Polynomial-based smooth trajectory planning for fruit-picking robot manipulator. *Information Processing in Agriculture*.

[7] Farhadi A., Redmon J. (2018). Yolov3: an incremental improvement. *Computer Vision and Pattern Recognition*.

[8] Ren S., He K., Girshick R., Sun J. (2015). Faster r-cnn: towards real-time object detection with region proposal networks. *Advances in Neural Information Processing Systems*.

[9] Liu W., Anguelov D., Erhan D. (2016). Ssd: single shot multibox detector. *Proc. European Conference on Computer Vision*.

[10] Lin T. Y., Goyal P., Girshick R., He K., Dollár P. Focal loss for dense object detection.

[11] Bochkovskiy A., Wang C. Y., Liao H. Y. M. (2020). Yolov4: optimal speed and accuracy of object detection. https://arxiv.org/abs/2004.10934.

[12] Zhang W., Chen K., Wang J., Shi Y., Guo W. (2021). Easy domain adaptation method for filling the species gap in deep learning-based fruit detection. *Horticulture Research*.

[13] Goodfellow I., Pouget-Abadie J., Mirza M. (2014). Generative adversarial nets. *Advances in Neural Information Processing Systems*.

[14] Zhu J. Y., Park T., Isola P., Efros A. A. Unpaired image-to-image translation using cycle-consistent adversarial networks.

[15] Yi Z., Zhang H., Tan P., Gong M. Dualgan: unsupervised dual learning for image-to-image translation.

[16] Kim T., Cha M., Kim H., Lee J. K., Kim J. Learning to discover cross-domain relations with generative adversarial networks.

[17] Mo S., Cho M., Shin J. (2018). Instagan: instance-aware image-to-image translation. https://arxiv.org/abs/1812.10889.

[18] Chen Y., Xia S., Zhao J. (2020). Appearance and shape based image synthesis by conditional variational generative adversarial network. *Knowledge-Based Systems*.

[19] Liang X., Zhang H., Xing E. P. (2017). Generative semantic manipulation with contrasting gan. https://arxiv.org/abs/1708.00315.

[20] Roy P., Häni N., Isler V. (2019). Semantics-aware image to image translation and domain transfer. https://arxiv.org/abs/1904.02203.

[21] Wu W., Cao K., Li C., Chen Q., Chen C. L. Transgaga: geometry-aware unsupervised image-to-image translation.

[22] Zhao Y., Wu R., Dong H. (2020). *Unpaired Image-to-Image Translation Using Adversarial Consistency Loss[C]//European Conference on Computer Vision*.

[23] Kim J., Kim M., Kang H., Lee K. (2019). U-gat-it: unsupervised generative attentional networks with adaptive layer-instance normalization for image-to-image translation. https://arxiv.org/abs/1907.10830.

[24] Nizan O., Tal A. Breaking the cycle-colleagues are all you need.

[25] Gokaslan A., Ramanujan V., Ritchie D., Kim K. I., Tompkin J. Improving shape deformation in unsupervised image-to-image translation.

[26] Sohn K., Zhang Z., Li C. L., Zhang H., Lee C. Y., Pfister T. (2020). A simple semi-supervised learning framework for object detection. https://arxiv.org/abs/2005.04757.

[27] Zhou Q., Yu C., Wang Z., Qian Q., Li H. Instant-Teaching: An End-to-End Semi-Supervised Object Detection Framework.

[28] Liu Y. C., Ma C. Y., He Z. (2021). Unbiased Teacher for Semi-Supervised Object Detection. https://arxiv.org/abs/2102.09480.

[29] Zoph B., Ghiasi G., Lin T. Y. (2020). Rethinking pre-training and self-training. *Advances in Neural Information Processing Systems*.

[30] Wang K., Cai J., Yao J., Liu P., Zhu Z. (2021). Co-teaching based pseudo label refinery for cross-domain object detection. *IET Image Processing*.

[31] Wang Z., Li Y., Guo Y., Fang L., Wang S. Data-uncertainty guided multi-phase learning for semi-supervised object detection.

[32] Ramamonjison R., Banitalebi-Dehkordi A., Kang X., Bai X., Zhang Y. Simrod: a simple adaptation method for robust object detection.

[33] Yang Q., Wei X., Wang B., Hua X. S., Zhang L. Interactive self-training with mean teachers for semi-supervised object detection.

[34] Wang H., Li H., Qian W. (2021). Dynamic pseudo-label generation for weakly supervised object detection in remote sensing images. *Remote Sensing*.

[35] Wang T., Yang T., Cao J., Zhang X. (2020). Co-mining: self-supervised learning for sparsely annotated object detection. https://arxiv.org/abs/2012.01950.

[36] Zhang W., Wang J., Liu Y. (2022). Deep-learning-based in-field citrus fruit detection and tracking. *Horticulture Research*.

[37] Koirala A., Walsh K. B., Wang Z., McCarthy C. (2019). Deep learning for real-time fruit detection and orchard fruit load estimation: benchmarking of ‘MangoYOLO’. *Precision Agriculture*.

[38] Isola P., Zhu J. Y., Zhou T., Efros A. A. Image-to-image translation with conditional adversarial networks.

[39] Wang Z., Simoncelli E. P., Bovik A. C. Multiscale structural similarity for image quality assessment.

